# A Thought for Parents

**Published:** 1857-02

**Authors:** 


A THOUGHT FOR PARENTS.—A New York daily inquires and replies: "Who are your aristocrats? Twenty years ago, this one made candles, that one sold cheese and butter, another butchered, a fourth thrived on a distillery, another was contractor on canals, others were merchants and mechanics. They are acquainted with both ends of society, as their children will be after them—though it will not do to say so out loud! For often you shall find that these toiling worms hatch butterflies—and they live about a year. Death brings a division of property, and it brings new financiers; the old gent is discharged, the young gent takes his revenues, and begins to travel—towards poverty, which he reaches before death, or his children do, if he does not. So that, in fact, though there is a sort of moneyed race, it is not hereditary; it is accessible to all: three good seasons of cotton will send a generation of men up—a score of years will bring them all down, and send their children to labor. The father grubs and grows rich, the children strut and spend the money. The children in turn inherit the price, and go to shiftless poverty; next their children, re-invigorated by fresh plebeian blood, and by the smell of clod, come up again."

